# Pharmacokinetics and Anti-Diabetic Studies of Gliclazide Nanosuspension

**DOI:** 10.3390/pharmaceutics14091947

**Published:** 2022-09-14

**Authors:** Sunitha Sampathi, Shubham Prajapati, Vijayabhaskarreddy Junnuthula, Sathish Dyawanapelly

**Affiliations:** 1 GITAM School of Pharmacy, GITAM Deemed to Be University, Rudraram, Hyderabad 502329, India; 2 Department of Pharmaceutics, National Institute of Pharmaceutical Education and Research (NIPER), Hyderabad 500037, India; 3 Drug Research Program, Faculty of Pharmacy, University of Helsinki, Viikinkaari 5 E, 00790 Helsinki, Finland; 4 Department of Pharmaceutical Science and Technology, Institute of Chemical Technology, Mumbai 400019, India

**Keywords:** Box–Behnken design, solubility, antisolvent precipitation, nanosuspension, quality by design, diabetes formulation

## Abstract

Gliclazide (GCZ), an antidiabetic medication, has poor solubility and limited oral bioavailability due to substantial first-pass metabolism. Thus, the purpose of the current study was to optimize and formulate a GCZ nanosuspension (NS) employing the antisolvent precipitation technique. A three-factor, three-level Box–Behnken design (BBD) was used to examine the impact of the primary formulation factors (drug concentration, stabilizer, and surfactant %) on particle size. The optimized NS contains 29.6 mg/mL drug, 0.739% lecithin, and 0.216% sodium dodecyl sulfate (SDS). Under scanning microscopy, the topography of NS revealed spherical particles. Furthermore, NS had a much better saturation solubility than the pure material, which resulted in a rapid dissolving rate, which was attributed to the amorphous structure and smaller particle size of the NS particles. Studies on intestinal permeability using the in vitro noneverted intestinal sac gut method (duodenum, jejunum, and ileum) and single-pass intestinal permeability (SPIP) techniques showed that the effective permeability was also increased by more than 3 fold. In the pharmacokinetic study, the C_max_ and AUC_0–t_ values of NS were approximately 3.35- and 1.9-fold higher than those of the raw medication and marketed formulation (MF). When compared to plain drug and commercial formulations, the antidiabetic efficacy of NS demonstrated that it had a significant impact on lowering glucose levels.

## 1. Introduction

The rate of dissolution of any active compound with poor water solubility ultimately determines the rate of absorption and, as a result, oral bioavailability [1]. As per the Biopharmaceutics Classification System (BCS), the dissolution of drugs belonging to Class II (low solubility and high permeability) is considered the rate-limiting phase, affecting the onset of action and intensity of pharmacological effects in vivo [2,3]. A second-generation sulfonylureas, Gliclazide (GCZ), [1-(3-azabicyclo (3, 3,0) oct-3-yl)-3-(p-tolylsulfonyl) urea] has transformed the treatment of type 2 diabetes/noninsulin dependent diabetes mellitus (NIDDM) [4]. It is a potential medication that has significant free-radical-scavenging activity in vitro and reduces the progression of diabetic retinopathy. GCZ has an uncertain and delayed absorption rate since it is a Class II medication with poor water solubility (0.19 mg/mL), along with poor wetting ability by water [5,6,7], resulting in considerable intra- and intersubject variability [8]. Because of the poor solubility of the drug from the standard formulation across the GIT membrane and substantial first-pass metabolism, the molecule has a slow absorption rate, making oral delivery difficult [2,9].

Several attempts have been undertaken to augment the solubility and dissolution rate of GCZ. These include solid dispersions prepared by various methods [10,11,12,13], complexation [14,15], ordered mixtures using water-soluble carriers such as mannitol and lactose [7], and micelles using cationic and anionic surfactants [5]. It was also reported that GCZ oral absorption is enhanced when it is suspended in polyethylene glycol 400 filled in soft gelatin capsules [16]. On the other hand, nanoparticles with chitosan, Eudragits for sustained release [17,18], floating alginate beads [19], and lipid-based nanoformulations [20] have been reported. Nanomedicine offers significant benefits in addressing bioavailability and targeting abilities [21,22,23,24,25]. Modified release tablets were reported to have an absolute bioavailability of 97% [26]. In situ micronization of GCZ using different stabilizers leads to quicker and thermodynamically stable dissolving crystals [6,27]. Nanocrystals and nanosuspensions (NS) were also reported [28,29]. Various studies have reported that GCZ solubility increases by various mechanisms, resulting in increased wettability with less particle size and converting the drug to an amorphous state. However, while all these techniques have certain advantages in terms of solubility, dissolution, drug loading, and bioavailability, many of them have significant drawbacks, such as the usage of costly specialist excipients, leakage of drugs, and scalability challenges that make them unsuitable. Furthermore, no previous research has examined the influence of formulation on drug absorption and intestinal permeability.

Preparing NS is another well-accepted method for enhancing drug solubility and dissolving rate in low-water-solubility medicines [30]. Furthermore, NS is effective in increasing medication bioavailability and decreasing interindividual variability, as well as fast-fed variability [31,32]. NS is a simple, scalable, and economical method of production. As a result, due to the significant advantages, converting the medication into NS (also known as nanocrystals) could be a promising option [33,34].

The present study aims to develop NS by a solvent–antisolvent precipitation method for improved bioavailability through enhanced drug dissolution. Herein, different stabilizers alone and in combination at different concentrations were explored by the design of experiments. To produce a stable system, the influence of drug and stabilizer concentrations, as well as sonication parameters, were investigated. Infrared spectrophotometry (FT-IR), Field emission scanning electron microscopy (FESEM), and the Differential Scanning Calorimetry (DSC) were used to examine the optimized formulation. In vitro dissolution, intestinal permeability, and pharmacokinetic studies were performed. Furthermore, antidiabetic activity was evaluated in male Wistar rats with normoglycemia.

## 2. Materials

GCZ was obtained from Yarrow Pharma Pvt. Ltd., Hyderabad, India. Sigma–Aldrich^®^, Mumbai, India, provided D-α-tocopherol polyethylene glycol 1000 succinate (TPGS), poloxamer-188 (pluronic F-68), solutol HS, and lecithin. BASF (Shanghai, China) supplied polyvinyl alcohol (PVA) and hydroxypropyl methylcellulose (HPMC E5). The SR life sciences of India provided sodium dodecyl sulfate (SDS) and povidone K-30 (PVP K-30). SD Fine Chem. Ltd. in Mumbai, India provided sucrose and polysorbate (Tween^®^80). Acetonitrile (ACN), trifluoroacetic acid (TFAA), and acetone were obtained from Merck, India. Trehalose and mannitol were purchased from TCI Chemicals in India. Egluna 40 (GCZ) marketed formulation, manufactured by Trinveni, Hyderabad, India. All additional reagents employed were of pharmaceutical quality, including methanol, ethanol, acetone, and mannitol.

## 3. Methods

### 3.1. Solvent–Antisolvent Precipitation Technique for GCZ NS

The NS was made using the solvent–antisolvent precipitation method, which was slightly modified from the literature [35]. GCZ was dissolved in acetone (40 mg/mL) to form an organic phase (OP). The polymers (SDS 0.25% and lecithin 1% *w*/*v*) were added to distilled water to prepare the anti-solvent phase (AP). In a nutshell, using a syringe, the organic phase (1 mL) was injected quickly into 10 mL of anti-solvent. (22 needle gauze) with magnetic stirring at 1000 RPM, and then the mixture was immediately ultrasonicated (8 s on and 4 s off) with a 35 W amplitude (Sonics & Materials, Inc., Newtown, CT, USA, Vibracell, 750) in a cold environment. Following ultrasonic treatment, the organic solvent was completely evaporated for 4 h while stirring continuously at 1000 RPM. The prepared NS was freeze-dried (Skadi-Europe; Model no: FD5508) for long-term storage. Trehalose (1% *w*/*v*) was added as a cryoprotectant during lyophilization.

### 3.2. Formulation by Design (FbD) Approach

A systematic examination of the consequences of variables on the final preparation is desirable. Henceforth, quality by design (QbD), a scientific method with predefined goals, is used to formulate GCZ-loaded NS. Candidates are encouraged to employ QbD in product development by drug regulatory agencies such as the US Food and Drug Administration (USFDA), Therapeutic Goods Administration (TGA), Medical and Healthcare Products Regulatory Agency, and others. From both industry and academia, this has generated much attention [36]. The predefined objectives, according to ICH Q8, include prior knowledge of risk, design of experiments (DoE), and handling of data across the entire product life cycle (R2). In comparison to traditional methodologies, QbD allows for a greater understanding of the process while also ensuring product quality at a lower cost [37].

The concept “formulation by design (FbD)” has replaced the word “quality by design (QbD)” regarding formulation development. Similar to QbD, FbD is similar, except that instead of focusing on critical material characteristics or attributes (CMAs), critical formulation attributes (CFAs) are considered.

The FbD process includes creating a quality targeted product profile (QTPP), categorizing critical process parameters (CPPs), key quality attributes (CQAs), and critical success factors (CFAs), in addition to risk analysis. To generate a design space, the screening factors are examined using an experimental design (optimization). The design space, a multidimensional pattern and interplay of variables, defines the zones that are possible and not feasible. Furthermore, operating within a design environment is not deemed a shift by regulatory authorities [38,39].

#### 3.2.1. Defining the QTPP

Setting up the QTPP, commonly referred to as the “goal or objective setting,” is the first phase in FbD. The QTPP’s definition includes a list of goals and ideal characteristics that, if attained, guarantee the product’s excellence, security, and efficiency.

#### 3.2.2. CQA Identification

CQAs are traits or attributes that, when maintained within a predetermined range, guarantee product quality. As a result, the next stage in the FbD-based method is to identify CQAs.

#### 3.2.3. CFAs and CPP Identification

This step includes identifying potential formulation and process parameters that could affect the selected CQAs. CFAs are formulation-related factors that influence CQAs, while CPPs are process-related factors.

#### 3.2.4. Prescreening Studies

CQAs can be influenced by a variety of elements, and considering all of them throughout the design process can be time-consuming. As a result, the one factor at a time (OFAT) technique was initially utilized to screen the factors to lessen the impact of components and enhance the ability of statistical design to predict outcomes.

#### 3.2.5. The Proportion of Organic Phase (OP) to Antisolvent Phase (AP) Optimization

The initial stage is to screen the organic-phase-to-antisolvent-phase ratio, because it has the greatest influence on the size of the particle and its distribution. The organic phase volume was held constant (1 mL) with the antisolvent phase volume varying from 10 to 40 mL. The effect of the ratio on particle size and Polydispersity index (PDI) was assessed. The impact of the ratio on the PDI and particle size was evaluated.

#### 3.2.6. Screening of Stabilizers

For a formulation to be stable at the nanoscale, stabilizers play a crucial role, and since there is no easy way to choose, a method based on a trial-and-error procedure using individual/combinations of different stabilizers was used [40]. Utilizing the OFAT approach, stabilizers were assessed at different concentrations for particle size, PDI, and stability (as determined by ocular inspection) [34].

#### 3.2.7. Experimental Design

The methodical approach to determining the impact of input elements (CFAs and CPPs) on the CQA is known as the design of the experiment. The pertinent parameters were optimized using response surface approaches, specifically the Box–Behnken design (BBD). The BBD is a type of incomplete block and factorial design that minimizes the sample size needed for coefficient estimation. It is thought to be more cost-effective than central composite designs [39].

To assess the influence of the independent variables (A) concentration of drug (mg/mL), (B) amount of stabilizer (%), and (C) level of surfactant (%) on the dependent variable (X) particle size, a three-level, three-element BBD design was used, as indicated in Table 1. Employing Design Expert^®^ software, response surface assessment was performed using contour (2D) and response surface plots (Version 13, Stat-Ease Inc., Minneapolis, MN, USA).

#### 3.2.8. Search for Optimized Preparation

A desirability function was used to optimize the search for the best possible formulation. The attractiveness value, which ranges from 0 to 1, is calculated based on the target values specified. The higher the value is, the more certain the desired results are. In addition, the design space was used to perform graphical optimization [41].

#### 3.2.9. Design Validation

Checkpoint analysis was used to validate the design. The results were compared to the projected values following the completion of three confirmatory trials.

### 3.3. Physicochemical Characterization of NS

#### 3.3.1. Particle Size, Polydispersity Index (PDI) and Zeta Potential (ZP)

Dynamic light scattering (DLS) analysis in a Malvern zeta sizer (Nano ZS, Malvern Instruments, UK) was used to determine the particle size, PDI, and ZP of NSs. Triple-distilled water was used as a dispersion medium, and the samples were diluted ten times before being analyzed on a Malvern Zeta sizer at 25 °C. All measurements were performed in triplicate, and the Z-average (d.nm) and PDI values were calculated. The ZP of the formulation was determined by dipping a palladium electrode in the diluted samples using a Malvern Zetasizer ZS (Nano series ZS 90, UK) [42].

#### 3.3.2. Lyophilization and Redispersibility Index (RDI)

For better stability and convenience of handling, the produced NS was subjected to lyophilization using a lab lyophilizer (Model no: Lab India FD5508). Cryo-chilled flasks were filled with samples to be lyophilized, along with the requisite amount of cryo-protectant. To obtain freeze-dried NS (FDNS), prefreezing with dry ice was used, followed by freeze-drying as per the reported procedure (−70 °C and 0.055 mbar pressure) [43]. The particle size of the FDNS was measured after they were thoroughly mixed with triple-distilled water. The redispersibility index (RDI) equation determines the NS redispersion potential [44,45,46].

(1) $$\mathrm{RDI}\;\left(\%\right)=\left[\frac{D_{0}}{D}\right]\times 100$$

D_0_ and D are the typical particle sizes before and after lyophilization, respectively.

#### 3.3.3. Scanning Electron Microscopy (SEM)

The surface morphology of pure drug and NS was imaged by SEM (QUANTA FESEM 250). SEM images were taken by mounting the sample over a double-sided adhesive carbon tape that was, in turn, mounted over aluminum pin stubs and sputter-coated with gold using an ion sputter before analysis. The samples were examined at a working distance of 10 mm with a 30 kV accelerating voltage and a magnification range of 500 to 120,000 times [47].

#### 3.3.4. Saturation Solubility

To screw vials holding 3 mL of different media, an excess of GCZ and freeze-dried NS were added individually (triple-distilled water, acetate buffer, phosphate buffer, and 0.1 N hydrochloric acid) and sonicated for 2 to 3 min to disperse the drug. The vials were maintained at 37 °C for 48 h on an incubator shaker (SI 300 UK) and centrifuged at 7500 rpm for 10 min. A Millipore 0.22 filter was used to separate and purify the clear liquid, and the GCZ concentration was evaluated using a UV–visible spectrophotometer set to 226 nm [48,49].

#### 3.3.5. In Vitro Release of GCZ NS

In USP type II equipment (LABINDIA, model no: DS 8000; Mumbai, India), a dissolution test of plain drug and NS of GCZ (equivalent amount of 40 mg) was performed. The samples were placed in a phosphate-buffered medium (pH 7.4; 900 mL). Throughout the experiment, a stirring rate of 100 rpm was used, and the temperature was held constant at 37 °C. Aliquots of 5 mL were removed and replaced with the same volume of fresh medium at predefined intervals. Filtrations of the samples were performed using nylon membrane syringe filters of 0.1 µm (Sigma–Aldrich), and drug concentration was evaluated using an established RP-HPLC method (Supplementary Method S1) [20]. The percentage of released drugs from bulk and NS was compared. The studies were carried out in triplicate, with the findings expressed as a percentage of the drug dissolved in pure drug and NS. The percentage release of the free drug and the formulation were compared, and the experiments were repeated three times.

#### 3.3.6. Stability Studies

The optimized freeze-dried GCZ-NS were stored at three different temperature conditions (5 ± 3 °C, 40 ± 2 °C and 25 ± 2 °C) for 6 months. At regular time intervals (0, 0.5, 1, 3, and 6 months), using a Malvern zeta-sizer, the samples were analyzed for particle size, PDI, and ZP.

### 3.4. Noneverted Intestinal Sac Study

With minor modifications, an in vitro noneverted intestinal sac investigation was carried out using previously reported procedures [50]. The Institutional Animal Ethical Committee (IAEC) certified the protocol (NIP/01/2018/PE/264). In summary, in the two groups, the male Wistar rats were separated at random (PD and GCZ NS), with three rats in each group. Following euthanasia, both groups of animals were treated with anesthetic ether. Surgical removal of the intestines followed by ice-cold saline washing was performed (50 mL). After separating the small intestine, a 5 cm ileum was separated in its place. The normal sacs (mucosal side) were filled with one mL of each sample containing PD and GCZ NS (1.3 mg), and both ends of the sac were ligated securely. For the entire study period, the PD dispersion and NS formulation-filled sacs were immersed into a beaker that contained 40 mL of PBS. (pH 7.4) with continual aeration and stirring at 100 rpm at 37 °C. The amount of drug that passed from the mucosal to the serosal side was measured by taking samples (3 mL) at specified time intervals (up to 120 min). The amount of medicament transferred from the mucosal to the serosal direction was determined using a designed RP-HPLC under identical column conditions at a wavelength of 228 nm.

The apparent permeability coefficient (Papp) was calculated using the equation below.

(2) $$\mathrm{Papp}=\mathrm{dQ}/\mathrm{dt}+1/\left(A+C_{0}\right)$$

where A is the surface area of the intestinal sacs, C_0_ is the initial concentration inside the sacs, and dQ/dt is the drug transport rate in the serosal medium.

### 3.5. In Situ Single-Pass Intestinal Perfusion (SPIP) Method

The invasive technique and SPIP study were completed as previously reported [50]. In short, the animals were split into 2 sets (PD and GCZ NS formulation), each with three animals. Thiopental sodium (50 mg/kg, intraperitoneal) was used to anesthetize the rats. The rats’ abdomens were incised 3–4.5 cm midline, and an ileal segment of approximately 10 cm length was separated using the ileocaecal intersection as a distal marker. At each end of the ileum, mid incisions were made, the lumen was washed with normal saline (37 °C), and both ends were cannulated with polyethylene tubing and ligated with silk suture. Then, using a syringe pump (Olives India), blank perfusion media (PBS pH 7.4) was pumped at a flow rate of 1 mL/min for 5 min. Later, the PD (dispersed in 0.5% *w*/*v* CMC) and NS formulation were infused at a continuous flow rate (0.2 mL/min) for 120 min. The ileal segment was covered with wet gauze soaked in isotonic saline throughout the experiment. The perfusate was collected every ten minutes at predefined periods and stored at −80 °C until analysis. The concentrations of drugs in perfusion samples were determined using RP-HPLC with a PDA detector set at a max of 228 nm (Supplementary Method S1). The computations were based on the outflow perfusate steady-state concentrations at the specified time points. A parallel tube model was used to calculate the steady-state intestinal effective permeability (Peff).

(3) Peff, rat=−Q·ln (Cout/Cin)/60·2πrl

where Q represents the perfusion rate (0.2 mL/min), r represents the radius of the intestinal segment (0.18 cm), and l represents the length of the intestinal segment (10 cm). Cin and Cout are the solute concentrations in the inlet and exit, corrected for fluid transfer.

### 3.6. Pharmacokinetic Studies

Male Wistar rats weighing 200 ± 20 g (4–5 weeks old) were used for the study and were supplied by the National Institute of Nutrition (NIN), Telangana, India. All animal studies were conducted according to “Guidelines for Care and Use of Laboratory Animals”, and the protocol was approved by the Institutional Animal Ethics Committee (IAEC) under protocol no NIP/01/2018/PE/264. The animals were acclimatized at a temperature of 20 ± 2 °C and relative humidity of 40–60% under natural light/dark conditions for one week before experiments. The animals were randomly distributed into 3 groups, each containing 6 animals. The plain drug (PD; dispersed in 0.5% *w*/*v* CMC), the GCZ NS formulation (6 mg/kg BW), and the marketed formulation (MF; 6 mg/kg BW) were all administered orally. Blood samples (250 µL) were collected from the retroorbital plexus into EDTA-coated tubes at definite time intervals (0, 15, 30, 60, 90, 120, 150, 180, 240, 300, 360, 420, 480, 540, and 600 min). Using an Eppendorf centrifuge, blood samples were centrifuged for 10 min at 7000 RPM. HPLC analysis (Supplementary Method S1) was used to process and analyze the separated plasma [51]. The Phoenix program Winnonlin version 6.3 (Pharsight, Certara company, USA) was used to compute pharmacokinetic parameters such as peak plasma concentration (C_max_), time to reach C_max_ (T_max_), and area under the curve (AUC) [52]. The data are presented as the mean and standard deviation.

### 3.7. In Vivo Antidiabetic Study

Rats with normoglycemia were separated into five groups (*n* = 6) and fasted overnight to test the antidiabetic activity of the formulations. Blank vehicles were provided to the control group (1 mL of 0.5% *w*/*v* CMC; p.o.), while the other groups were given 1 mL of a plain drug (PD; 6 mg/kg; p.o.), optimized NS (NS; 6 mg/kg; p.o.), and marketed formulation (MF; 6 mg/kg; p.o.). After 30 min of drug administration, each animal was administered a glucose overload (2 g/kg, p.o.). Blood samples were taken from the tail vein before drug delivery and at 30 min intervals for the next 12 h. Fasting blood glucose levels were checked using glucose-oxidase-peroxidase active strips (Accu-check kit strips; Roche Diagnostics, GmbH, Mannheim, Germany).

## 4. Results and Discussion

### 4.1. Formulation of GCZ NS

The method of solvent–antisolvent precipitation was used to make NS in this investigation. This approach entails dissolving a drug in an organic phase in an antisolvent phase containing a stabilizer, which causes rapid drug precipitation because of desolvation, resulting in nanosized drug particles. We used acetone as the solvent and water as the antisolvent phase.

According to the Ostwald–Mier theory, crystallization begins when the system achieves supersaturation, accompanied by nucleation and crystal development. When a saturated drug solution is added to an antisolvent, a supersaturated solution is formed; the solvent is evaporated, resulting in the production of many nuclei; and crystal development continues. Furthermore, the ice-cold condition is chosen because the drug’s solubility in the solvent combination is reduced at lower temperatures, resulting in higher supersaturation and slower diffusion. The Damkohler number (Da = T_mix_/T_ppt_), a ratio of mixing time to precipitation time, can be used to understand the process. If Da is greater than 1, the process is considered mixing-controlled, and the mixing time (T_max_) is larger than the precipitation time (Tppt). Because the time required to reach supersaturation is slow in this situation, larger particles are produced because of increased particle growth over nucleation. If Da < 1, then supersaturation occurs quickly, and nucleation takes precedence over crystallization if T_max_ is less than T_ppt._, resulting in a large number of nuclei with smaller particle sizes [53]. As a result, achieving Da < 1 is recommended to obtain smaller particle sizes. The use of ultrasonic waves may generally lessen the T_mix;_ nonetheless, stabilizers are employed to increase T_ppt_ [54,55]_._

### 4.2. FbD-Based Approach

#### 4.2.1. Defining the QTPP

Enhancing the solubility and bioavailability was the goal of the GCZ-loaded NSs. Therefore, regarding the formulation, the QTPP was well defined and is presented in Table 2.

#### 4.2.2. Identification of CQAs

The FbD approach’s fundamental step is recognizing the CQA [56]. QTPP is accomplished once the CQAs are recognized and accurately regulated within the limit. In the current experiment, particle size was selected as the CQA. The medication’s poor water solubility is improved by particle size reduction to the nanoscale [57]. Table 2 shows the CQAs that were chosen with explanations.

#### 4.2.3. Identification of CFAs and CPPs

CQAs can be affected by many factors, and an Ishikawa fish-bone diagram was used to show the cause-and-effect relationship in Supplementary Figure S1. Studies for prescreening were conducted to examine the connection between the variables and the response from a functional standpoint.

#### 4.2.4. Prescreening

Incorporating all elements into the design would result in a higher number of runs, further complicating the design. To determine the OP to AP ratio and the best stabilizer, prescreening experiments were conducted using the OFAT method. The process of nanoprecipitation involves critical variables; to explore the optimum conditions, we considered different formulation and process variables, such as the selection of the solvent system, solvent–antisolvent phase ratio, and type and concentration of stabilizer used. After adjusting one parameter at a time while keeping the other parameters constant, the effect of the aforementioned parameters on particle size and PDI was noticed.

#### 4.2.5. Selection of OP and Optimization of the OP-to-AP Ratio

**Figure 1:**
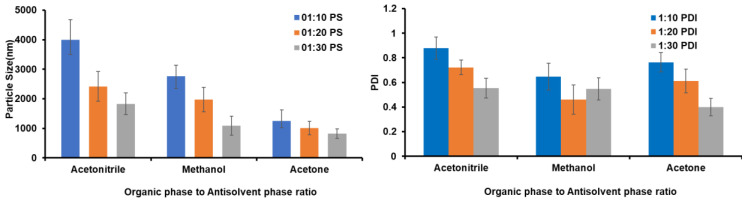
Effect of organic-phase-to-antisolvent-phase ratio on particle size and PDI.

Depending on the highest solubility of the drug, acetone, acetonitrile, and methanol were used as the organic phase (OP) in the preliminary trials. As the drug has low solubility, triple-distilled water was taken as the antisolvent phase (AP), and its miscibility with many solvents makes it an appropriate AP. An incorrect OP/AP ratio may cause nonuniform particle formation, resulting in unpredictable particle size distribution and aggregation. The effect of varying the OP-to-AP ratio on particle size and PDI is shown in Figure 1.

The NS prepared using a 1:30 ratio of OP:AP resulted in a lower particle size compared to other ratios (particle size of 1218 nm with a PDI of 0.434) without aggregation, and the system was uniform in terms of particle size and size distribution. The choice of appropriate solvent plays a key role in stabilizing the NS by regulating the number of crystal nuclei generated. Furthermore, GCZ exhibits a solubility of 49.6 mg/mL in acetone. A greater percentage of antisolvent reduces the drug’s ability to dissolve in the solvent at an optimal OP-to-AP ratio, which increases the nucleation rate and results in smaller particles. Furthermore, the availability of more antisolvents lengthens the diffusion length, slowing the expansion of produced nuclei [55].

#### 4.2.6. Selection of Stabilizer

Trial batches with a 1:30 OP/AP ratio and 30 mg of GCZ were used to determine the type of stabilizer. The particles in NS are very energetic and thermodynamically unstable and agglomerate or undergo Ostwald ripening to stabilize the system. The use of an appropriate stabilizer at a sufficient concentration lowers interfacial tension, inhibits crystal formation, and acts as a steric or electrostatic barrier between particles [33,58]. Preliminary studies with several stabilizers were conducted since it was important to optimize a suitable stabilizer and its concentration. [43].

**Figure 2:**
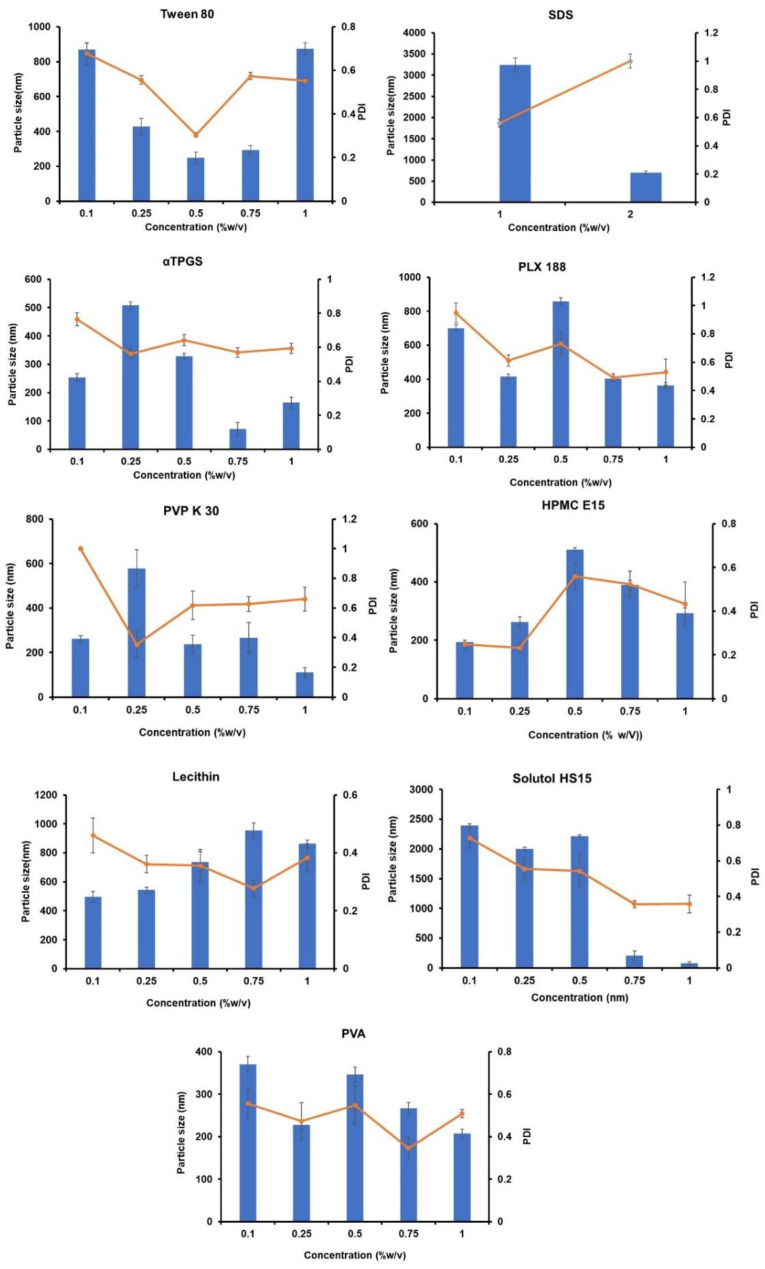
Selection of stabilizers for the preparation of gliclazide nanosuspension.

The average particle size and PDI attained with diverse stabilizers are shown in Figure 2. Individual stabilizers were tested at varying concentrations (0.1, 0.25, 0.5, 0.75%, 1.0% *w*/*v*), and further combinations were explored based on the results. According to the results, the particle size increased in the following order: TPGS > HPMC > PVA > PVP K-30 > Solutol HS > Poloxamer 188 > Tween 80 > Lecithin > SDS. Using nonionic surfactant stabilizers such as Tween 80 and Solutol HS produced a high particle size and PDI (>0.5) at various concentrations compared to polymeric stabilizers (HPMC E15, PVP-K30, PVA and Poloxamer 188), giving fewer particles but a high PDI (>0.5). HPMC E15 is a large molecule nonionic stabilizer that provides stability via steric stabilization with a very low particle size. The findings imply that the formation of large crystals is promoted by nonionic surfactants, which is related to a low supersaturation state and a limited amount of crystal nuclei. Similarly, the polymeric stabilizers resulted in low particle size compared to nonionic surfactants, but the very high PDI suggests that there was nonuniformity/heterogeneity in the prepared system.

HPMC is a polymer made up of methoxy and hydroxypropyl assemblies with a large hydrophobic part that attracts water and can establish hydrogen bonds with drugs. Although HPMC formulations reduced particle size, they also caused more aggregation and visible particle settling. The rationale given is that HPMC adds greater viscosity to the fluid, which may stifle the particle production process. The formulations made with PVP K-30 and PVA were found to be more homogeneous and transparent than those made with HPMC. However, after 4 h, the formulation was not stable, as indicated or seen by the settling of particles. This may be due to insufficient polymer adsorption onto the hydrophobic drug surfaces, and PVP is unable to provide sufficient surface energy in the stabilization of drug surfaces [59]. The highest particle size was observed in SDS at concentrations of 1.0% *w*/*v* and 2.0% *w*/*v*, giving particle sizes of 3243.86 ± 86.38 and 702.3 ± 26.99 with PDI values of 0.561 ± 0.11 and 1.000 ± 0.00, respectively. However, SDS at 0.5% *w*/*v* and above did not precipitate the system; instead, it helped solubilize the drug [60]. Even below the critical micelle concentration (CMC), oxyethylene blocks of T80, a nonsurfactant and small molecule ionic surfactant such as SDS, resulted in a considerable increase in drug solubility, resulting in a large particle size up to a definite concentration. [30].

TPGS, a nonionic surfactant, resulted in particle sizes ranging from 71.81 ± 43.33 to 507.96 ± 63.27 nm but showed a high PDI (>0.5). Similarly, lecithin, a natural stabilizer, gave a particle size ranging from 496.25 ± 36.96 nm to 954.23 ± 52.47 nm with low PDI (˂0.4). The more pronounced Ostwald ripening that occurs, the higher the PS levels. Meanwhile, Ostwald ripening can be avoided by using the right stabilizer combination. [61,62]. From earlier reports, it is understood that a single stabilizer will not help to form a homogenous NS with low PDI [63].

Hence, we studied the effect of a combination of surfactants (HPMC, SDS, PLX 188, lecithin, Tween 80, and PVA) in stabilizing the formulations to reduce Ostwald ripening. As an outcome, a variety of stabilizers were used to try to develop a stable formulation with lower particle size and PDI. Various combinations of stabilizers yielded different formulations (F1-F13), producing particle sizes in the range from 96.49 ± 15.00 to 4795.66 ± 26.84 nm and PDI values in the range from 0.326 ± 0.05 to 1.000 ± 0.00, as shown in Table 3. For example, the F7 formulation consisting of HPMC (0.1% *w*/*v*) and lecithin (0.1% *w*/*v*) displayed a particle size of 1399.66 ± 14.84 with a PDI of 0.920 ± 0.03, and the F10 formulation consisting of Tween 80 (0.1% *w*/*v*) and PVA (0.1% *w*/*v*) showed a particle size of 999.96 ± 281.36 with a PDI of 0.702 ± 0.16. From the findings, it was noted that the F12 formulation consisting of SDS (0.25% *w*/*v*) with lecithin (1% *w*/*v*) surfactants displayed a particle size of 96.49 ± 15.00 nm and PDI of 0.326 ± 0.05. SDS, a small molecule that is an ionic surfactant that acts as an electrostatic stabilizer, helps stabilize the system, as it has a zeta potential of −22 mV. Because it is adsorbed on the surface of the particles, lecithin acts as a steric stabilizer, preventing aggregation and resulting in a stable system. Lecithin is a common pharmaceutical excipient that has no known side effects [64,65]. Hence, a combination of stabilizers was selected for further study. Combining stabilizers may provide appropriate surface-active electrostatic and steric stabilization for the systems and is also favored for long-term stabilization [66].

#### 4.2.7. Design of Experiments

For the study, there were 15 runs (with three center points), as shown in Supplementary Table S1. Using multiple linear regression, polynomial models such as linear, quadratic, and two-factor interaction (2FI) were created. The predicted R^2^, adjusted R^2^, and coefficient of variance were used to choose the models (CV).

Size plays a critical role in improving the solubility of poorly soluble drugs. Smaller particles have a greater surface area, wherein the saturation solubility is increased. Therefore, one of the CQAs was chosen to be particle size for producing a GCZ-loaded NS. The particle size ranged from 90.22 to 146.12 nm after 15 well-prepared trials. This implied that the quadratic model was substantial with a negligible lack of fit. The final model F value was 39.40, indicating a 0.01% likelihood that it was caused by noise. The R^2^ and corrected and anticipated R^2^ values were 0.9861, 0.9611, and 0.8288, respectively. The model’s sufficient precision was 19.986, greater than the necessary number of 4, demonstrating the ability to explore the design area [67].

The model terms A, C, AC, A^2^, B^2^, and C^2^ all had *p* values of less than 0.05, showing that they had a major influence on the response. These terms were now regarded as significant, and the resulting regression equation was

(4) $$\mathrm{Particle}\;\mathrm{size}=92.93+15.59A-12.77C-14.22\mathrm{AC}+7.24A^{2\;}+10.02B^{2}+6.18C^{2}$$

**Figure 3:**
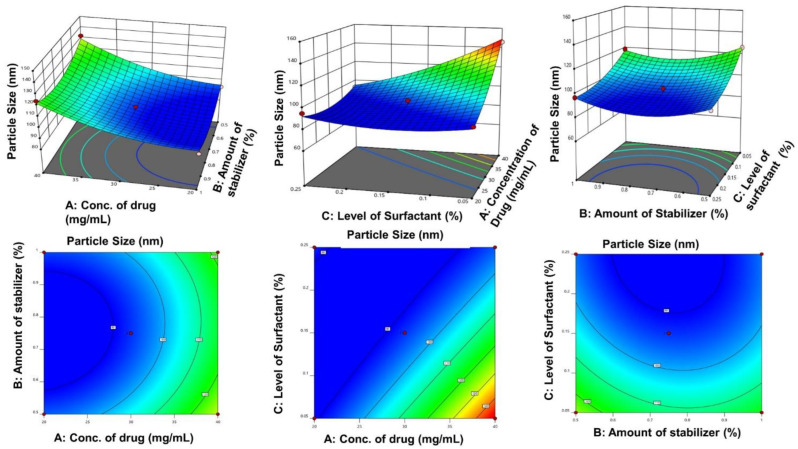
Surface response and contour plots with respect to the particle size.

At both lower and higher stabilizer-to-drug ratios, the particle size increased, as shown in the response surface plot (Figure 3). For coating newly developed surfaces, low stabilizer concentrations might not be sufficient to prevent nuclei diffusion, resulting in larger particles.

According to Alexandridis et al., below the threshold micellar temperature of 25 °C, a larger stabilizer ratio produces multilayers, and as the layer thickness increases, so does the particle size [68].

#### 4.2.8. Search for Optimized Formulation

**Figure 4:**
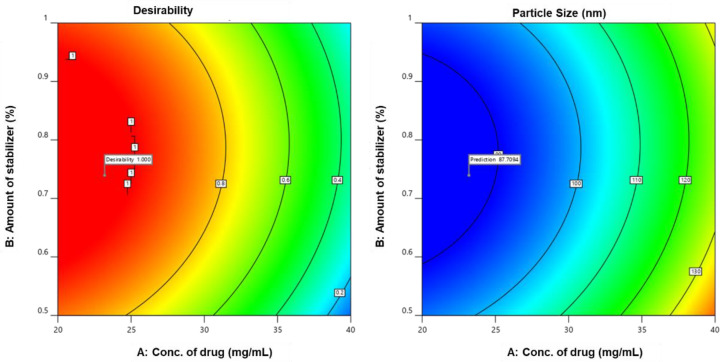
Design space and validation.

The solutions offered by the design were assigned a desirability value by numerical optimization using the desirability function. The solution with the greatest desirability of 1 was designated as the optimized formulation F_opt_. A drug concentration of 29.6 mg, a stabilizer amount of 0.735%, and a surfactant level of 0.216% were used for optimization. For further graphical improvement, the target values of CQAs, such as reduced particle size, were limited. A design space was developed, as shown in Figure 4.

#### 4.2.9. Validation of the Design

Validation by three checkpoint formulations was used to determine the model’s correctness and resilience. The predicted mean value for size was 86.98 nm (Supplementary Table S2), but the observed mean value for size was 87.12 nm. The outcomes of these formulations matched the values that the software had predicted, proving the validity of the model [69].

### 4.3. Physicochemical Characterization of NS

#### 4.3.1. Particle Size, PDI and ZP

The particle size, PDI, and zeta potential of the optimized NS were assessed utilizing a Malvern zeta sizer immediately after dilution (1:10) with Milli-Q water. The resulting formulation displayed an average particle size of 96.49 ± 15 nm with a PDI of 0.326 ± 0.05. Since the PDI of the developed formulation was less than 0.3, NS had a consistent particle size distribution and homogeneity [70]. The physical stability of NS can be estimated using the zeta potential, and the optimized formulation has a ZP of −22 ± 5.6 mV. The surface charge on nanoparticles can result through ionization of the particle surface or surfactant adsorption, both of which help to stabilize the NS [71].

#### 4.3.2. Lyophilization and Redispersity Index (RDI)

Lyophilization was used to improve solid-state characterizations and make them easier to handle. Furthermore, the cryoprotectant for freeze-drying is commonly utilized in NS before solidification, which can be employed to protect NS against solidification damage. Cryoprotectants are frequently employed to fill the spaces between the Nanocrystal (NC) following the elimination of water during lyophilization to prevent irreversible aggregation and maintain the redispersibility of NS [72].

**Figure 5:**
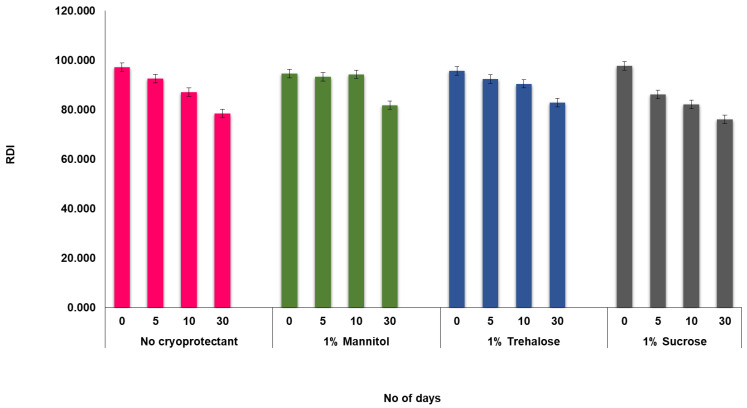
Effect of various cryoprotectants on the redispersity index (RDI) over 30 days.

At a concentration of 1% *w*/*v*, mannitol, trehalose, and sucrose are three different types of cryoprotectants that were studied. The particle size and PDI of the lyophilized powders were measured at room temperature for one month. Figure 5 shows the RDI of the lyophilized NS. The RDI did not change significantly after one month with and without cryoprotectants [73].

#### 4.3.3. Scanning Electron Microscopy

The topography of the plain drug and formulation is shown in Supplementary Figure S2. The morphology of unprocessed drugs showed an irregular shape with a particle size in µm and a wide particle size distribution with discrete units, while NS formulated with solvent–antisolvent precipitation transformed the drug into uniformly sized nanoparticles (50–100 nm). The impact of nanosizing on particles upon antisolvent precipitation to generate many nuclei while preventing crystal formation was revealed in this work, in [74].

#### 4.3.4. Saturation Solubility

Saturation solubility studies of GCZ and GCZ-NS were performed in different media to determine the increase in solubility of the drug after preparing NS, and the information is provided in Table 4. The NS of GCZ showed an increase in solubility compared to the GCZ plain by 14-fold as NS in triple-distilled water and acetate buffer, 6-fold in 0.1 N hydrochloric acid, and 4-fold in phosphate buffer. When comparing plain GCZ to NS formulations, enhanced solubility was observed in all media. This could be due to the large surface area of the nanosized particles, the drug’s decreased crystallinity, and the surfactants’ improved wettability [75].

#### 4.3.5. In Vitro Release of GCZ NS

**Figure 6:**
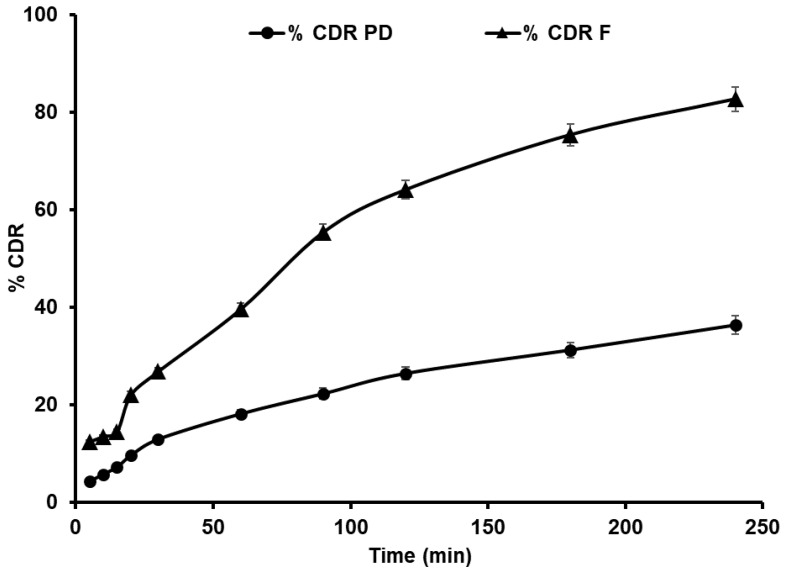
In vitro dissolution profiles in phosphate buffer (pH 7.4; mean ± SD; *n* = 3) of the plain drug (PD) and formulation (F).

A USP apparatus type II was used to determine the % cumulative drug release in phosphate buffer (pH 7.4) medium under sink conditions. Figure 6 depicts the drug release characteristics of the plain drugs and NSs. The plain drug dispersion displayed 12.91 ± 1.22% and 36.36 ± 2.23% release within 30 and 240 min, respectively. Furthermore, the formulation displayed 26.81 ± 2.46% and 82.67 ± 3.82% within 30 and 240 min, respectively. As supported by other authors, the increased availability of dissolved GCZ and drug nanoparticles may have resulted in increased drug release [6,7]. When compared to the pure drug, the NSs had a faster rate of dissolution. The Noyes–Whitney/Nernst–Brunner equation explains this, stating that a decrease in particle size results in an increase in surface area to the nano range, favoring an increase in dissolution [76]. Particle size, shape, state (amorphous or crystalline), and habit (cubic or spherical) are a few of the physical traits that control a drug’s solubility and dissolution rate under physiological conditions. The current research is focused on a micron-sized, cubic-shaped crystalline pure medication with poor solubility that makes dissolving extremely slow. Dissolution augmentation by NS may result from (a) an amorphous nature translation (shown by DSC, Figure S5, Supplementary Method S3; XRD, Figure S6, Supplementary Method S4), (b) the drug and stabilizer forming hydrogen bonds (confirmed by FTIR, Figure S4, Supplementary Method S2), (c) particle size reduction from the micron to the nanometer range (size measurements), and (d) particle shape (as observed by SEM, Figure S2). All of these elements contribute to the solubility and dissolving properties of the drug under study [71].

#### 4.3.6. Stability Studies

For a total of 6 months, GCZ-NS stability experiments were conducted at three different temperatures (0, 0.5, 1, 3, and 6 months). Particle size, PDI, and ZP were examined concerning the influence of stability conditions, and the data are included in Table 5. Storage under refrigerated conditions (5 ± 3 °C) increased the particle size from 87.12 ± 3.76 (0th day) to 102.14 ± 16.28 (6th month). At a high temperature of 40 ± 2 °C, an increase in size was observed from 0.5 to 6 months from 119.42 ± 5.21 nm to 212.38 ± 8.04 nm. When the formulation was stored at 25 ± 2 °C, the particle size increased slightly from 96.92 ± 8.31 nm (0.5 months) to 153.92 ± 5.73 nm (6th month). The drastic increase in particle size at high temperature may be because the stabilization with time data lost its integrity. Because of Ostwald ripening, the lower surface coverage would lead to an increase in particle size [77].

### 4.4. Noneverted Intestinal Sac Permeation Study

The drug permeability between various colonic sections (duodenum, ileum, and jejunum (proximal region)) was calculated and displayed against time points. In different segments, the mean apparent permeability (Papp) for PD in the duodenum, jejunum, and ileum was 0.56 × 10^−4^ cm/s, 0.66 × 10^−4^ cm/s, and 0.28 × 10^−4^ cm/s, respectively. In the case of the NS formulation, the mean apparent permeability in the duodenum, jejunum, and ileum was found to be 0.87 × 10^−4^ cm/s, 0.91 × 10^−4^ cm/s in the jejunum, and 0.98 × 10^−4^ cm/s. The formulation demonstrated superior results to the PD with an apparent permeability enhanced by 1.5-, 1.37-, and 3.53-fold in the duodenum, jejunum, and ileum, respectively. Compared to the PD, drug absorption in NS was improved. In comparison to the duodenum, maximum absorption occurs in the lower intestine. The permeability of active substances across the rat stomach can be measured using the noneverted sac model and can forecast in vivo human absorption, in addition to numerous in vitro approaches [78]. The findings of this study imply that drug administration by way of nanoparticles can improve mucosal permeability by reducing size, which leads to greater drug particle penetration and, in turn, improved drug absorption throughout the colon [79].

### 4.5. In Situ Single-Pass Intestinal Perfusion Method (SPIP)

Despite encouraging in vitro outcomes, most drugs do not act in vivo for a variety of reasons, including low absorption, water-insoluble materials, and unstable physical properties. The gut mucosa is the main obstruction controlling the absorption process. To determine formulation efficiency in an intact rat model, we conducted the SPIP investigation. The permeability of PD and NS in the rat ileum was tested. Effective permeability (Peff) was determined using the collected perfusate’s steady-state drug concentrations. The results revealed that the drug’s effective permeability improved from 0.072 ± 0.002 × 10^−4^ to 0.36 ± 0.04 × 10^−4^ cm/s. The foremost benefit of the in situ SPIP approach is that it allows for complete physiological circumstances in the experimental animals. This approach, which is based on the local absorption rate across the epithelial barrier, aids in the prediction of intestinal absorption in humans. Our findings indicated that drug delivery in NS forms increased intestinal permeability because of elements such as compact particle size, larger surface area, more solubility, and improved dissolving. This approach aids in human absorption prediction. Intestinal permeability refers to a compound’s capacity to travel over the intestine’s epithelial barrier. It is an accurate reflection of the transport velocity through the epithelial barrier and a direct measurement of the local absorption rate. Due to characteristics such as smaller drug size, greater surface area, improved solubility, and improved dissolution, our findings suggest that drug administration in NS increased intestinal permeability [34].

### 4.6. Pharmacokinetic Studies

**Figure 7:**
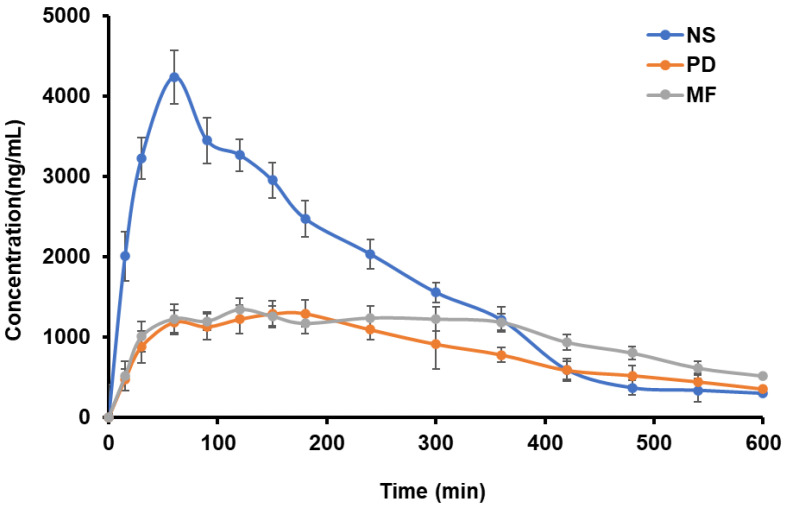
Pharmacokinetic profiles in male Wistar rats (*n* = 6) of the drug in plasma following oral administration of plain drug suspension and NS formulation.

The plasma drug concentration versus time profile of PD, GCZ NS, and MF after oral administration is shown in Figure 7. Table 6 lists the pharmacokinetic parameters. The chromatogram is shown in Supplementary Figure S3. The GCZ NS concentration maximum (C_max_) and area under the curve (AUC_0–t_) were approximately 3.35 and 1.9 times higher than the PD. Since the drug belongs to BCS class II and has low solubility, a higher dissolving rate via NS aids in reaching a higher C_max_ than a PD. The increased bioavailability of the drug was primarily due to two mechanisms. First, nanosizing reduced the particle size while increasing the surface area. Second, the thickness of the diffusion layer was reduced, and the adhesion surface area between nanoparticles and the intestinal epithelium of villi was increased, resulting in direct contact between the surfaces. Third, the drug was released immediately, making it more available at the absorption site [30]. This is in agreement with the enhanced bioavailability of GCZ previously reported with lipid nanoparticles [80] and cubosomes [81].

### 4.7. In Vivo Antidiabetic Study

**Figure 8:**
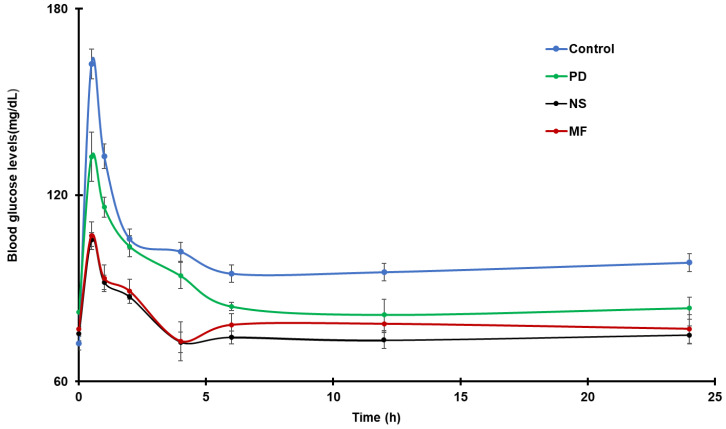
Anti-diabetic activity of GCZ formulations in normoglycemic Wistar rats after oral administration (6 mg/kg, *n* = 6).

Following the delivery of pure GCZ and NS to Wistar rats, the mean blood glucose levels (mg/dL) are reported in Figure 8. The results showed that rats given NS had significantly improved biological activities compared to animals given PD. The decrease in glucose levels can be connected to GCZ’s effective solubility in NS form, which allows for faster and more complete absorption [30]. In a recent study, oral administration of gliclazide-loaded mucilage microparticles showed a hypoglycemic effect in diabetic rabbits, and the results of the present study are in good agreement with published reports [82].

## 5. Conclusions

The antisolvent precipitation approach was used in this study to suggest a novel formulation of weakly water-soluble gliclazide as NS. Particle size was greatly influenced by process and formulation parameters. The BBD design was used to investigate the impact of factors on responses, and then numerical and graphical optimization was used to find the best formulation. The optimized formulation contains SDS and lecithin. The nanoparticles were found to be amorphous, as determined by DSC. As seen in the SEM images, the uneven cubic micro-range form changed to nanosized particles. Because of the amorphous nature and smaller particle size of GCZ NS, the drug release % was much higher than that of pure drug. NS showed improved penetration across the intestinal mucosa in in vitro and in vivo investigations compared to PD. The C_max_ and AUC_0–t_ values of NS were approximately 3.35- and 1.9-fold higher than those of the plain drugs in an in vivo study. The study found that using GCZ NS to improve solubility and thus bioavailability in vivo is a faster, less expensive, and more effective method. The crystallinity of the drugs plays an important role in establishing their solubility; however, we did not include it in the CQAs or stability studies. Further studies are required to understand this observation.

## Figures and Tables

**Table 1:** Factors for the design of the experiment.

	Independent Variables	Levels		
		Low (−1)	Medium (0)	High (+1)
A	Concentration of drug (mg/mL)	20	30	40
B	Amount of stabilizer (%)	0.5	0.75	1
C	Level of surfactant (%)	0.05	0.15	0.25
Responses		Constraints		
X	Particle size	Minimize		

**Table 2:** QTPP and CQA selection and justification.

QTPP	Target	Justification
Formulation	Nanosuspension (NS)	The solubility and bioavailability can be improved by NS formulation
Route of administration	Oral	The commercial formulation is oral, and we are working to increase oral bioavailability
Dissolution	Higher compared to plain drug	Increased solubility could result in accelerated dissolution
Pharmacokinetics	Should be better than the already available form	For increased bioavailability
Stability	No visible signs of aggregation/cake formation up to 120 days after formulation	The efficiency of the formulation depends on particle size. It is vital to maintain the same stability
**CQAs**		
**CQA**	**Target**	**Justification**
Particle size	nm	The solubility and dissolution are both increased when size is reduced to the nanoscale because it increases surface area. Bioavailability is improved via higher solubility and dissolution

**Table 3:** Data of particle size and PDI with a combination of stabilizers.

Formulation	Concentration of Stabilizers (% *w*/*v*)							
	HPMC	SDS	PLX 188	Lecithin	Tween 80	PVA	Avg. PS (nm)	Avg. PDI
F1	0.1	0.1	-	-	-	-	825.7 ± 89.38	0.831 ± 0.06
F2	0.25	0.25	-	-	-	-	2038.66 ± 17.00	0.769 ± 0.39
F3	0.5	-	0.1	-	-	-	4777.66 ± 29.48	0.873 ± 0.21
F4	0.1	0.25	-	-	-	-	4795.66 ± 26.84	1.000 ± 0.00
F5	0.1	-	0.1	-	-	-	475.30 ± 64.00	0.619 ± 0.12
F6	0.1	-	0.5	-	-	-	1018.83 ± 58.38	0.896 ± 0.08
F7	0.1	-	-	0.1	-	-	1399.66 ± 14.84	0.920 ± 0.03
F8	-	0.25	-	0.1	-	-	939.33 ± 72.34	0.654 ± 0.32
F9	-	-	-	-	1	1	247.3 ± 130.44	0.347 ± 0.09
F10	-	-	-	-	0.1	0.1	999.96 ± 281.36	0.702 ± 0.16
F11	0.1	-	-	-	0.5	-	1134.1 ± 158.66	0.697 ± 0.08
F12	-	0.25	-	1.0	-	-	96.49 ± 15.00	0.326 ± 0.05
F13	-	0.1	-	1.0	-	-	146.70 ± 2.55	0.386 ± 0.01

**Table 4:** Saturation solubility.

Different Medias	Plain Drug (GCZ) (µg/mL)	NS Formulation (µg/mL)
Water	49.6 ± 6.37	681.87 ± 63.24
0.1 N Hydrochloric acid (pH 1.2)	29.61 ± 9.58	182.05 ± 10.36
Acetate buffer (pH 4.5)	24.88 ± 6.58	364.18 ± 89.31
Phosphate buffer (pH 7.4)	629.04 ± 163.05	2604.57 ± 321.68

**Table 5:** Short-term stability study of the NS formulation under different conditions with respect to particle size, PDI, and ZP.

Temperature	Months	PS (nm)	PDI	ZP (mV)
5 ± 3 °C	Initial	87.12 ± 3.76	0.172 ± 0.026	−22.19 ± 2.16
	0.5	89.86 ± 5.56	0.265 ± 0.022	−19.17 ± 1.78
	1	90.65 ± 5.48	0.271 ± 0.042	−21.42 ± 2.95
	3	92.88 ± 8.13	0.289 ± 0.021	−19.86 ± 2.42
	6	102.14 ± 16.28	0.308 ± 0.038	−21.84 ± 3.01
25 ± 2 °C	Initial	87.12 ± 3.76	0.172 ± 0.026	−21.56 ± 2.86
	0.5	96.92 ± 8.31	0.297 ± 0.028	−22.19 ± 2.16
	1	124.71 ± 6.18	0.263 ± 0.029	−22.99 ± 2.52
	3	149.52 ± 6.98	0.304 ± 0.032	−22.66 ± 2.65
	6	153.92 ± 5.73	0.322 ± 0.025	−23.12 ± 2.38
40 ± 2 °C	Initial	87.12 ± 3.76	0.172 ± 0.022	−22.19 ± 2.16
	0.5	119.42 ± 5.21	0.297 ± 0.020	−21.02 ± 3.90
	1	133.26 ± 7.39	0.303 ± 0.025	−20.04 ± 3.12
	3	180.66 ± 6.98	0.327 ± 0.030	−20.38 ± 2.90
	6	212.38 ± 8.04	0.322 ± 0.032	−20.65 ± 3.44

**Table 6:** Pharmacokinetic parameters of the drug (6 mg/kg) in Wistar rats following oral administration of plain drug suspension and NS formulation (mean ± SD; *n* = 6). * F_rel_ calculated for the average AUC values.

PK Parameter (Units)	Plain Drug Suspension	NS	MF
AUC_(0–t)_ (ng/mL·h)	8334.106 ± 102	16,766.277 ± 125	10,163.584 ± 132
AUC_(0–α)_ (ng·h/mL)	10,238.84 ± 105	19,649.178 ± 128	12,627.599 ± 135
C_max_ (ng/mL)	1290.813 ± 118	4234.691 ± 120	1346.013 ± 115
T_max_ (h)	3.00	1.00	2.00
K_el_ (1/h)	5 ± 0.04	0.104 ± 0.06	0.209 ± 0.01
t_1/2_ (h)	3.744 ± 1.2	6.662 ± 0.15	3.316 ± 2.5
% Relative Bioavailability (F_rel_) *	100.00	201.175	121.951

## Data Availability

The authors declare that the article materials of this study are available within the article, and additional information is available from S.S. upon request.

## Supplementary Materials

The following supporting information can be downloaded at: https://www.mdpi.com/article/10.3390/pharmaceutics14091947/s1, References [83,84] are cited in the supplementary materials. Figure S1: Schematic Ishikawa fish-bone diagram for the formulation of nanosuspension; Table S1: Design of experiments (DOE) for the preparation of GCZ NS; Table S2: Validation data for the optimized NS formulation; Figure S2. SEM images of GCZ PD (A) and GCZ NS (B); Figure S3: Chromatogram of gliclazide in serum; Figure S4: AT-FTIR overlay of GLZ-PD, Lecithin, SDS, Physical mixture, GLZ + Lecithin, GLZ+ SDS and NS Formulation; Figure S5: Overlay of DSC thermograms of (A) PD—blue line; (B) Physical mixture—red line; and (C) NS—black line. Figure S6: XRD Pattern of the plain drug (GCZ) (A) and GCZ NS formulation (B). S1. HPLC analysis; S2. ATR-FTIR spectroscopy; S3. Differential scanning calorimetry (DSC); S4. XRD.
