# Supplementary material for: Pharmacokinetics and Anti-Diabetic Studies of Gliclazide Nanosuspension
Source: Pharmaceutics. 2022 Sep 14;14(9):1947. doi: 10.3390/pharmaceutics14091947 (PMC9500859; doi:10.3390/pharmaceutics14091947)
Supplement: Supplementary file 1 [file pharmaceutics-14-01947-s001.zip › pharmaceutics-1874172-supplementary.pdf]

Pharmacokinetics and Anti-Diabetic Studies of Gliclazide Nanosuspension

Sunitha Sampathi [1,2,*,†], Shubham Prajapati [2,†], Vijayabhaskarreddy Junnuthula [3,*] and Sathish Dyawanapelly [4,*]

[1] GITAM School of Pharmacy, GITAM Deemed to Be University, Rudraram, Hyderabad 502329, India
[2] Department of Pharmaceutics, National Institute of Pharmaceutical Education and Research (NIPER),
Hyderabad 500037, India
[3] Drug Research Program, Faculty of Pharmacy, University of Helsinki, Viikinkaari 5 E, 00790 Helsinki, Finland
[4] Department of Pharmaceutical Science and Technology, Institute of Chemical Technology, Mumbai 400019, India
* Correspondence: ssampath@gitam.edu (S.S.); junnuthula.vijayabhaskarreddy@helsinki.fi (V.J.); sa.dyawanapelly@ictmumbai.edu.in (S.D.)
† These authors contributed equally to this work.

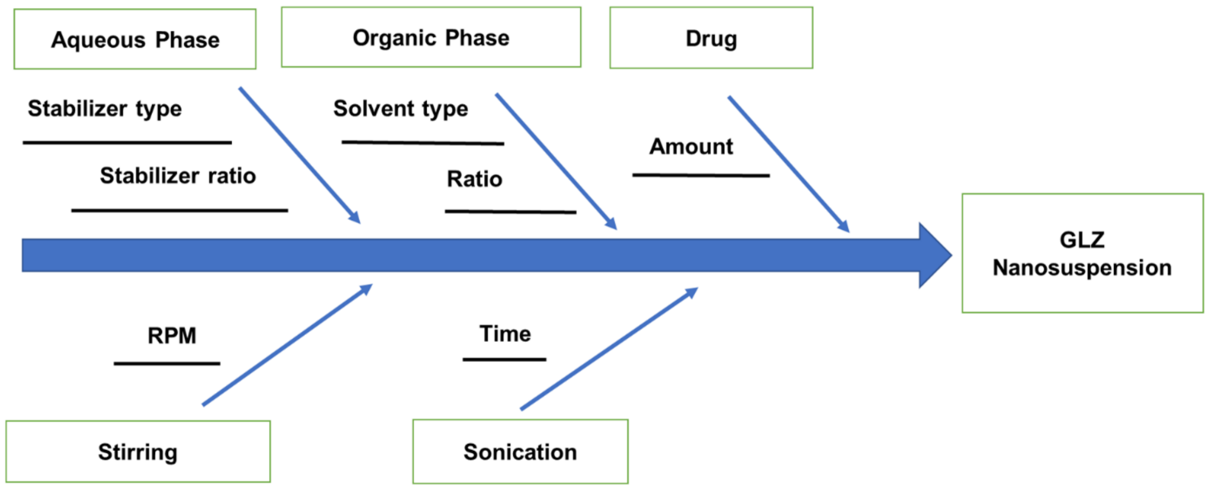

Figure S1: Schematic Ishikawa fish-bone diagram for formulation of nanosuspension

Table S1: Design of experiments (DOE) for the preparation of GCZ NS

RUN	A	B	C	X
1	20	1	0.15	91.1
2	20	0.75	0.05	91.26

3	30	1	0.25	96.68
4	40	0.5	0.15	132
5	30	0.5	0.25	95.18
6	40	0.75	0.25	92.99
7	30	0.75	0.15	90.22
8	40	1	0.15	124.32
9	30	0.5	0.05	126
10	20	0.5	0.15	93.32
11	30	1	0.05	118.64
12	40	0.75	0.05	146.12
13	30	0.75	0.15	96.14
14	20	0.75	0.25	95
15	30	0.75	0.15	92.42

Note: A,B, C: Independent variables X: Dependent variable.

Table S2: Validation data for the optimized NS formulation.

**S. No**	**Factors**			**Particle size (nm)**		
	A	B	C	Observed	Predicted	% Bias
1	29.60	0.739	0.216	87.12±2.6	86.98	14 %
2	29.8	0.75	0.102	100.24±3.2	99.97	27.03 %

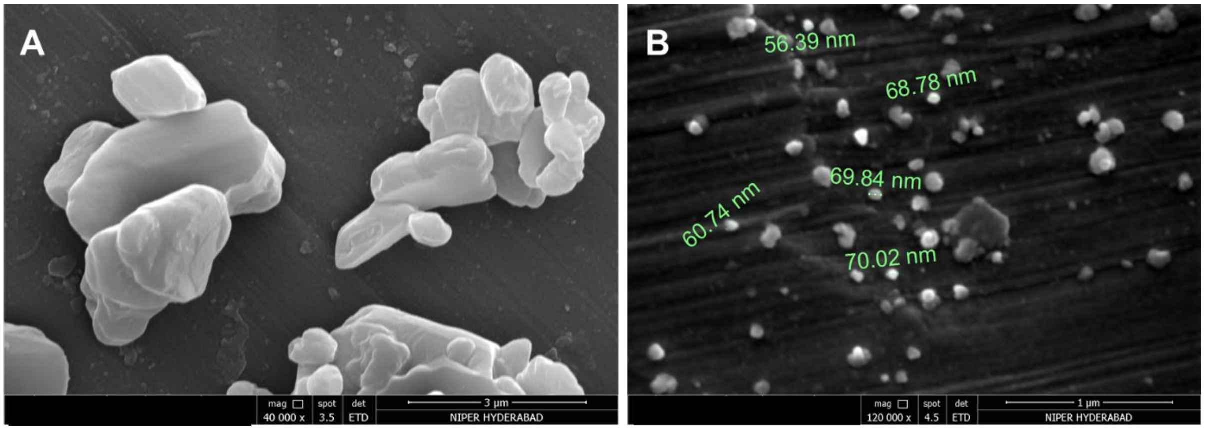

Figure S2: SEM images of GCZ PD (A) and GCZ NS (B)

Supplementary Method:

S1: HPLC analysis

Using chromatographic separation, analysis was performed using previously reported method(1).

S1.1 Preparation of standards

The initial stock at 1 mg/mL was made by weighing GCZ and glibenclamide (internal standard). A secondary stock of 100 g/mL was prepared from this and a standardization curve in the region of 0.250 - 64 μg/mL was performed. The devised method was accredited for various factors including the limit of detection(LOD), the limit of quantification(LOQ), linearity, accuracy, precision, specificity, recovery and robustness(1).

S1.2. Sample extraction for bioanalysis

The drug was extracted from plasma using the protein precipitation technique(1). In nutshell acetonitrile (250 μL) was added to rat plasma (50 μL) and vortex blended to ensure effective extraction. The supernatant was analyzed at a λ max of 228 nm by HPLC after subjecting the samples to centrifugation at 8500 rpm for 10 minutes. The HPLC method was established using the parameters listed above and the procedure adopted as per the reported publication (2). Signal to noise ratios (S/N) for LOD and LOQ concentrations are greater than 3.3 and 10, respectively. The LOD and LOQ in this instance were 0.0625 and 0.250 g/mL, respectively. The drug was found to be linear with a regression coefficient (R^2) of 0.999 in the range of 0.250 to 64 g/mL. The results of the precision and accuracy checks were used to establish the sample quality assurance. Analyte concentrations retrieved from the biological matrix are used to assess the response to the answer of the pure standard to determine the percent recovery (Figure S3).

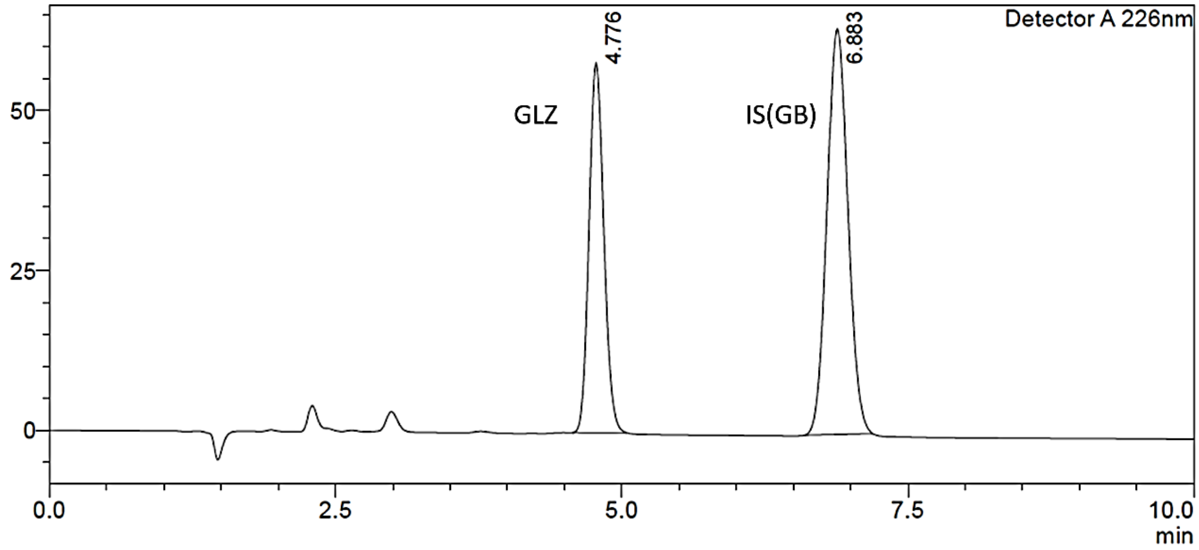

Figure S3: Chromatogram of gliclazide in serum

S2 ATR-FTIR Spectroscopy

ATR-FTIR spectrum was performed by using a Perkin (Elmer) spectrophotometer. The ATR-FTIR analysis was conducted to check any interaction of chemical bonds between drugs and excipients. The pure drug(GLZ), lecithin, SDS, physical mixture of drug with lecithin, physical mixture of drug with SDS, Physical mixture (drug, lecithin, SDS) and freeze-dried nanosuspension were examined at wave numbers between 4000-450 cm^{-1} with 1.0 cm^{-1} resolution (3).

IR spectra of excipients, formulations, and plain GCZ were captured to reveal component compatibility, as displayed in figure S4. Scan resolutions varied from 4000 to 400 cm^{-1}. The pure drug peaked characteristically at 3270, 2932, 1707, 1596, and 1349 cm^{-1}. The presence of identifiable peaks in the physical mixture and freeze-dried NS with the

absence of fresh peaks indicate that there is no chemical interaction between the medication and the stabilizer (Figure S4) (1).

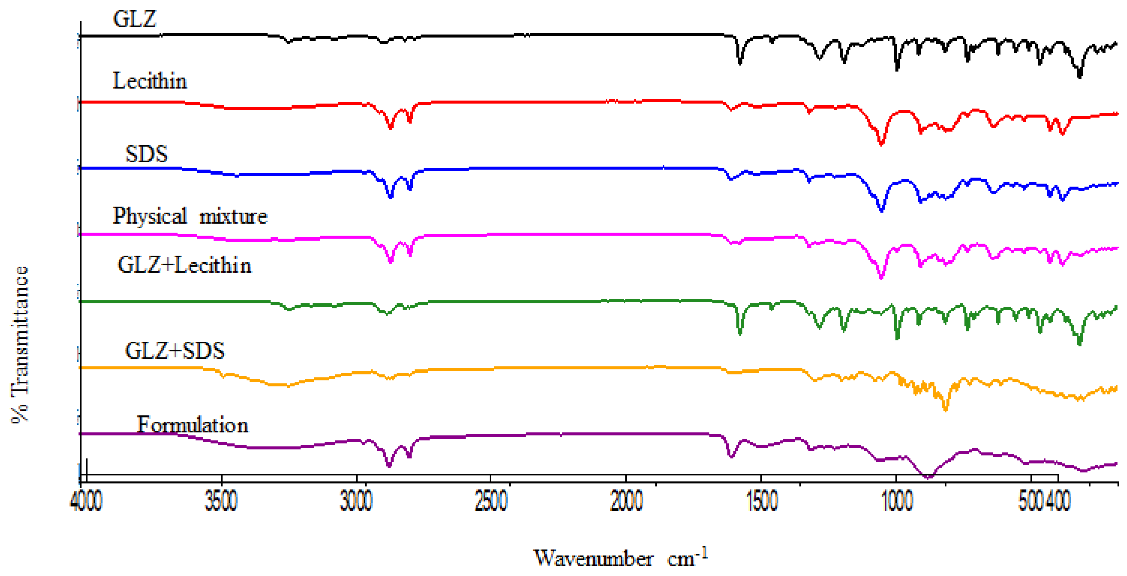

Figure S4: AT-FTIR overlay of GLZ-PD, Lecithin, SDS, Physical mixture ,GLZ + Lecithin, GLZ+ SDS & NS Formulation

S3. Differential scanning calorimetry (DSC)

DSC was utilized to determine the physical nature and possibility of any chemical interactions between the drug and excipients used. Thermograms of GLZ and NS formulation was obtained using a DSC calorimeter (DSC Shimadzu, DSC 60, Kyoto, Japan) at a heating rate of 5ºC/min at a heating range of 200ºC under a nitrogen atmosphere(4,5).

The results of a DSC examination of a pure drug, a physical mixture, and optimized nanosuspensions are presented in Figure S5. At 170°C, the pure drug (GLZ) displayed a pronounced endothermic peak (corresponding to the melting point of the drug). Both

peaks were present in the physical mixture, but the drug peak was shifted from 170 to 194.8°C, which could be owing to drug–stabilizer interaction. However, optimized nanosuspensions revealed no endothermic peak matching the drug, indicating that the drug was likely converted to amorphous form, whereas a cryoprotectant peak was discovered at its melting point. As a result of DSC, the crystalline drug is converted to an amorphous state. This could be because a polymer coat forms over the drug particle, functioning as a crystal development inhibitor.

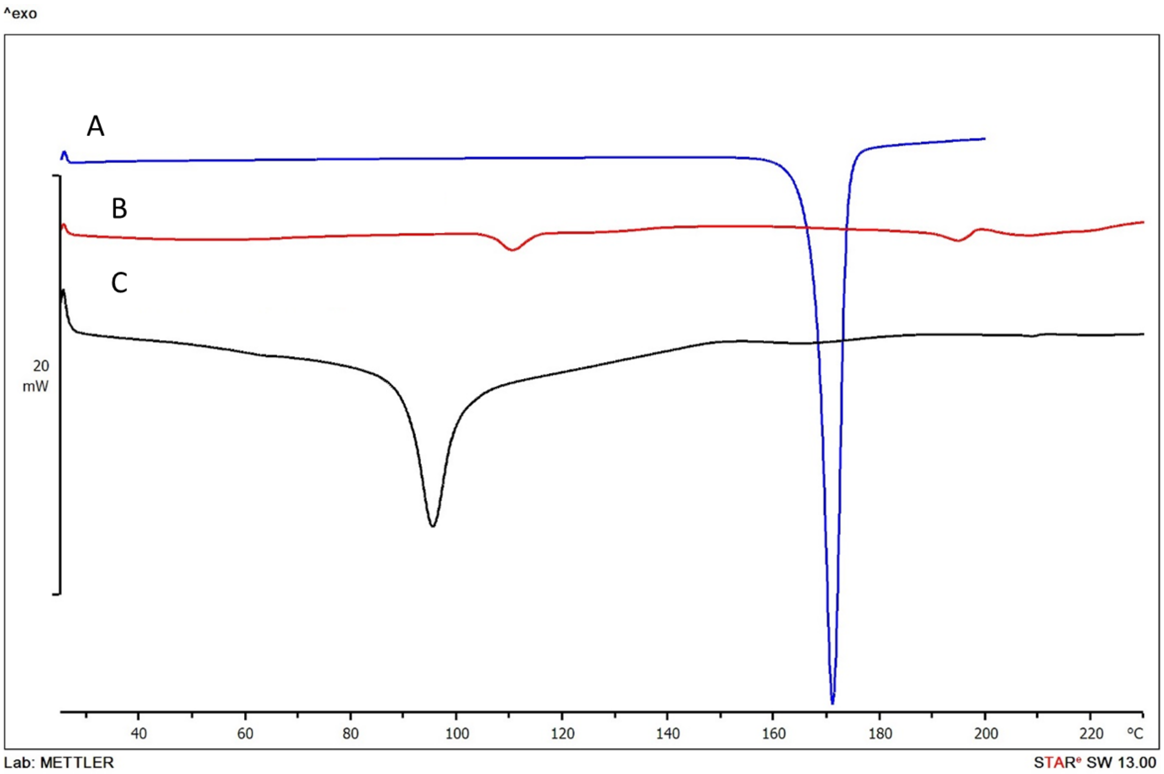

Figure S5: Overlay of DSC thermograms of A) PD-blue line; B) Physical mixture-red line; C) NS-black line.

S4. X-ray diffraction (XRD)

In x-ray studies an automatic x-ray diffractometer (Rigaku Dmax 2500, Rigaku, Tokyo, Japan) equipped with an x-ray generator was used. Nickel filtered Cu kα1 radiation having a wavelength of 1.5406 Å, operating at 35 kW and 20 mA in the range (2θ) of 20 to 80 degrees was used. X-ray diffractograms for drug, physical mixture and for NS were obtained at a scanning rate of 1 degree (2θ) per minute.

Results and Discussion:

XRD is very much helpful for identifying polymorphic changes in the drug during formulation processing as the XRD graph act as fingerprint for each and every drug and its polymorphs. It is widely used for analyzing whether the solid is crystalline or amorphous. The XRD patterns of gliclazide exhibits sharp peak at 2θ scattered angle 10.57,16.86,18.53,23.54 and corresponding peak intensities of 633,452,362,296 respectively. This indicates crystalline nature of drug. The peak intensity for formulation were also measured at same 2θ scattered angle of 10.7,18.25,23.61 and corresponding linear counts were found to be 18,15,12. Based on the peak intensities it shows that the degree of crystallinity was reduced in presence of stabilizer.

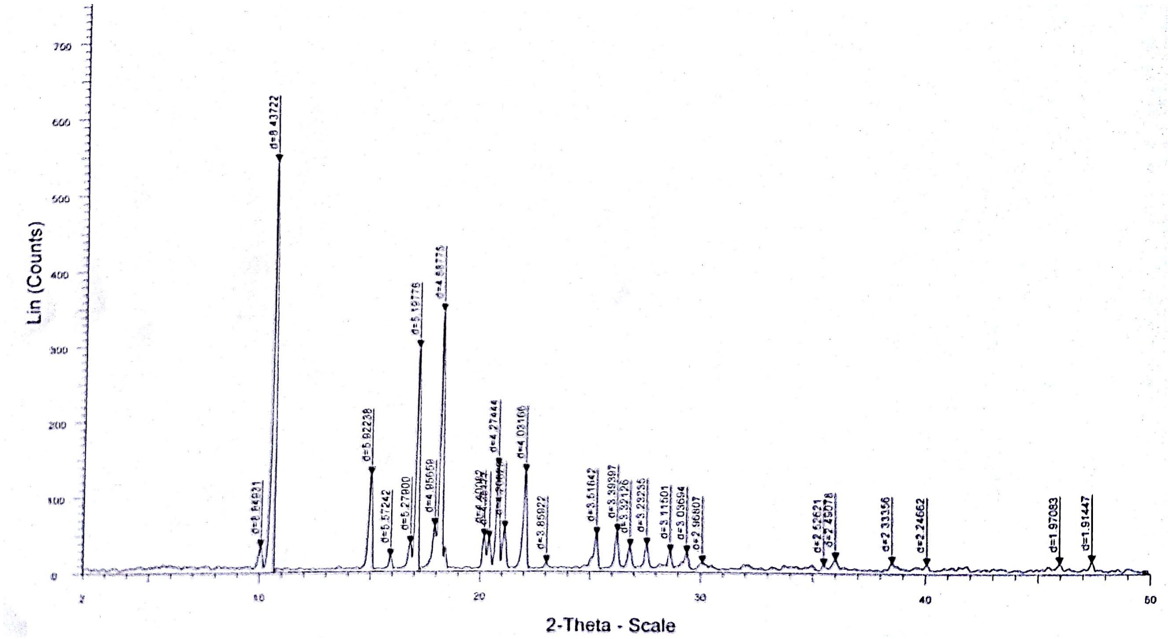

(A)

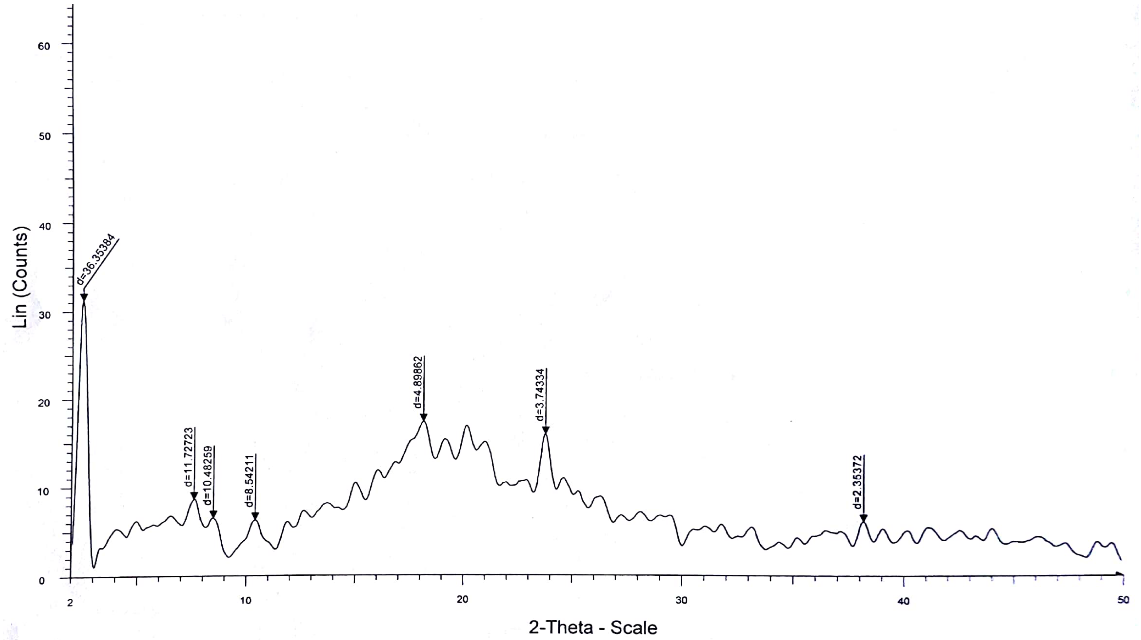

(B)

Figure S6: XRD Pattern of the Plain drug (GCZ) (A) and GCZ NS formulation (B)

References:

1. Patel P, Pailla SR, Rangaraj N, Cheruvu HS, Dodoala S, Sampathi S. Quality by Design Approach for Developing Lipid-Based Nanoformulations of Gliclazide to Improve Oral Bioavailability and Anti-Diabetic Activity. AAPS PharmSciTech. 2019 Feb;20(2).
2. Gera S, Talluri S, Rangaraj N, Sampathi S. Formulation and Evaluation of Naringenin Nanosuspensions for Bioavailability Enhancement. AAPS PharmSciTech. 2017 Nov;18(8):3151–62.
3. Afifi SA, Hassan MA, Abdelhameed AS, Elkhodairy KA. Nanosuspension: An emerging trend for bioavailability enhancement of etodolac. Int J Polym Sci. 2015;2015.
4. Karakucuk A, Celebi N, Teksin ZS. Preparation of ritonavir nanosuspensions by microfluidization using polymeric stabilizers: I. A Design of Experiment approach. Eur J Pharm Sci. 2016 Dec;95:111–21.
5. Rangaraj N, Pailla SR, Shah S, Prajapati S, Sampathi S. QbD aided development of ibrutinib-loaded nanostructured lipid carriers aimed for lymphatic targeting: evaluation using chylomicron flow blocking approach. Drug Deliv Transl Res. 2020;10(5):1476–94.
